# Effect of a phase 2 cardiac rehabilitation program on obese and non-obese patients with stable coronary artery disease

**DOI:** 10.1186/s43044-020-00119-4

**Published:** 2021-01-07

**Authors:** Ahmed El Missiri, Walaa Adel Abdel Halim, Abdo Saleh Almaweri, Tarek Rashid Mohamed

**Affiliations:** Cardiology Department, Faculty of Medicine, Ain Shams University, Abbassia Square, Abbasia, Cairo, 11566 Egypt

**Keywords:** Cardiac rehabilitation, Cardiac rehabilitation program, Coronary artery disease, Obese, Obesity, Exercise training

## Abstract

**Background:**

Obesity is associated with significant cardiovascular morbidity and mortality effects. Cardiac rehabilitation programs cause a significant reduction in cardiovascular mortality and a reduction in all cardiovascular risk factors. Up to 80% of patients referred to cardiac rehabilitation programs are either overweight or obese. This study aimed to compare the effects of a phase 2 cardiac rehabilitation program on obese and non-obese patients with stable coronary artery disease following total revascularization by coronary angioplasty.

**Results:**

This was a prospective study including 120 patients with stable coronary artery disease. Patients were enrolled in a 12-week phase 2 cardiac rehabilitation program. Patients were classified into two groups based on their body mass index (BMI): those with a BMI < 30 kg/m^2^ were considered non-obese (*n* = 58) while those with a BMI ≥ 30 kg/m^2^ were considered obese (*n* = 62). At baseline, BMI and blood pressure (BP) were recorded; fasting blood sugar, triglyceride levels, total cholesterol, high-density lipoprotein cholesterol (HDL-C), and low-density lipoprotein cholesterol (LDL-C) levels were assessed; and echocardiography was used to measure left ventricular ejection fraction (LVEF). These were re-assessed after completion of the program. At baseline, there were more females in the obese group 20 (32.25%) vs 6 (10.13%) (*p* = 0.04), more hypertensives (*p* = 0.023), and less smokers 32 (51%) vs 46 (79%) (*p* = 0.025). Obese patients achieved fewer metabolic equivalent of tasks (METs) 7.97 ± 2.4 vs 9.74 ± 2.47 (*p* = 0.007) and had higher LDL-C levels 121.63 ± 36.52 mg/dl vs 95.73 ± 31.51 mg/dl (*p* = 0.005). At the end of the program, obese patients showed more reduction in BMI − 1.78 ± 1.46 kg/m^2^ vs − 0. 60 ± 0.70 kg/m^2^ (*p* < 0.001) and systolic and diastolic BP (*p* = 0.016 and 0.038, respectively). LDL-C level was more reduced in the obese group − 25.76 ± 14.19 mg/dl vs − 17.37 ± 13.28 mg/dl (*p* = 0.022). Non-obese patients had more increase in LVEF (*p* = 0.024). There was no difference between obese and non-obese patients in the magnitude of increase in METs achieved (*p* = 0.21).

**Conclusion:**

Cardiac rehabilitation programs lead to an improvement in cardiovascular disease risk factors with more reduction in BMI, BP, and LDL-C levels in obese patients compared to non-obese ones. LVEF was more increased in non-obese individuals. Exercise capacity in the form of METs achieved was equally improved in both groups.

## Background

Obesity, an indicator of total adiposity, is a well-known risk factor of cardiovascular disease and is associated with an increased incidence of cardiovascular morbidity and mortality. Obesity is associated with an increased incidence of hypertension, insulin resistance, dyslipidemias, left ventricular hypertrophy, and an unfavorable sedentary lifestyle. Additionally, it has been linked to an increase in circulating inflammatory mediators with alterations in fibrinolysis and coagulation which may lead to endothelial dysfunction and atherosclerosis [1–4].

There is an ongoing global trend of an increase in the number of overweight and obese individuals among children, adolescents, and adults. The average global prevalence of obesity among adults is nearly 19% with an additional 39% being overweight. Prevalence varies from one country to another with the combined incidence of being overweight and obese in Egypt and other Middle Eastern countries being among the highest in the world ranging from 74 to 86% in females and 69 to 77% in males according to reports from the World Health Organization [5–7].

Cardiac rehabilitation is the provision of educational and lifestyle-modifying interventions combined with prescribed exercise training to patients suffering or recovering from different forms of cardiovascular diseases or interventions such as coronary artery disease and heart failure, following percutaneous coronary revascularization, cardiac surgery, heart transplantation, and cardiac device implantation [8–11].

Cardiac rehabilitation programs for coronary artery disease patients have been shown to reduce cardiovascular mortality by nearly 24%. Patients participating in such programs benefit from significant morbidity benefits such as reduction of blood pressure and body weight, improving functional capacity, controlling blood lipid and blood glucose levels, and helping patients resume their normal physical, social, and vocational status following recovery from a myocardial infarction (MI) or acute coronary syndrome [12–14].

Cardiac rehabilitation is divided into three phases: phase 1, cardiac rehabilitation starts during hospitalization for an index cardiovascular event and serves as an introduction to cardiac rehabilitation; phase 2, cardiac rehabilitation which is a medically supervised in-hospital program that usually starts 1 month following an index cardiovascular event and is considered the most important phase of cardiac rehabilitation; and phase 3, cardiac rehabilitation which refers to the long-term activities and lifestyle modifications a patient performs independently out-of-hospital [8–12].

This study aimed to compare the effect of a phase 2 cardiac rehabilitation program on obese and non-obese patients with stable coronary artery disease following total revascularization by coronary angioplasty.

## Methods

The study protocol was approved by the Institutional Ethical Committee, and an informed consent was obtained from each participant. This was a prospective study performed in the period from July 2016 to March 2017 on 120 patients with stable coronary artery disease who had complete coronary revascularization by coronary angioplasty performed in the 12 months before enrollment in the study and were on optimal guideline-directed medical therapy.

Subjects were classified into obese and non-obese patient groups. A patient was considered obese if their body mass index (BMI) was more than or equal to 30 kg/m^2^ while they were considered non-obese if their BMI was less than 30 kg/m^2^. BMI was calculated using the following formula: BMI = weight (kg)/height (m^2^) [15].

All patients were enrolled in the phase 2 cardiac rehabilitation program offered at our institution. The presence of any of the following criteria led to patient exclusion from the study: exertional angina, significant coronary stenosis on angiography, recent (less than 3 months) myocardial infarction or acute coronary syndrome, left ventricular ejection fraction (LVEF) less than 40%, recent (less than 3 months) hospitalization for decompensated heart failure, prior participation in a cardiac rehabilitation program, a physical or mental disability that would impede exercise training, having a heart rhythm other than sinus rhythm, hypertrophic cardiomyopathy, more than mild pulmonary hypertension, more than moderate valvular stenosis or regurgitation, prior implantation of a permanent pacemaker or other cardiac implantable electric device, significant peripheral arterial disease, any acute illness, chronic renal impairment, a history of transient ischemic attacks or cerebrovascular stroke, and chronic obstructive airway disease. Additionally, patients were excluded from the study if they had missed more than three sessions of exercise training during the study period or if any of the previously mentioned exclusion criteria developed after inclusion in the study.

Doses of lipid-lowering, anti-hypertensives, and hypoglycemic medications are needed to remain unchanged for 2 weeks before enrollment in the program and during the whole length of the study period. If a patient required a change in the doses of such medications, they were excluded from the study.

### Clinical and anthropometric assessment

Thorough history taking and clinical examination were performed for all subjects. Weight and height were measured to calculate BMI. Resting blood pressure was recorded using an appropriately sized cuff where an average of three measurements taken 2 min apart with the patient seated having their back supported and their feet resting on a flat surface according to current practice guidelines [16, 17]. Current smoking status was assessed. Patients were assessed for the presence of coronary artery disease risk factors, namely hypertension, diabetes mellitus, and dyslipidemia.

### Laboratory blood tests

Two antecubital venous blood samples were obtained from each patient: one after 8 h of fasting and the other after 12 h of fasting. The first sample was used to measure fasting blood sugar levels and the latter to measure total cholesterol, triglyceride, and high-density lipoprotein cholesterol (HDL-C) levels using enzymatic methods. Low-density lipoprotein cholesterol (LDL-C) was calculated using the Friedewald formula: LDL-C (mg/dl) = total cholesterol (mg/dl) − HDL-C (mg/dl) − 1/5 of triglycerides (mg/dl) [18].

### Trans-thoracic echocardiography

Standard trans-thoracic echocardiography was performed for all patients in the left lateral decubitus position using a Vivid S5 machine (GE Vingmed, Netherlands) equipped with an M4S matrix array probe with a frequency range of 1.7–3.5 MHz. Echocardiography was performed by an echocardiographer accredited by the European Association of Cardiovascular Imaging.

From the parasternal short-axis view at the level of the papillary muscles, left ventricular end-diastolic diameter (LVEDD) and left ventricular end-systolic diameter (LVESD) were measured following the practice guidelines. Left ventricular ejection fraction (LVEF) was measured using the modified biplane Simpson’s method of discs from the apical 4- and 2-chamber views [19].

### The cardiac rehabilitation program

All patients were enrolled in the program provided at the cardiac rehabilitation unit in our institution which is a 12-week twice-weekly (24 sessions) comprehensive program with progressive prescribed treadmill exercise training being its cornerstone.

Patients were assessed at baseline using a symptom-limited treadmill exercise test according to the modified Bruce protocol to identify their peak heart rate [20]. During that test, the metabolic equivalent of tasks (METs) achieved was automatically recorded by the treadmill. One MET was defined as the amount of oxygen consumed while sitting at rest which equals 3.5 ml of oxygen per kilogram of body weight per minute [21].

The program is run by a group of cardiologists, nutritionists, psychiatrists, trained nurses, and physiotherapists.

The program provides dedicated educational sessions and handouts covering the diagnosis of coronary artery disease, its risk factors, and the importance of risk factor modification; smoking cessation; the importance of adherence to medications; nutritional guidance and counseling; stress management; and psychological counseling sessions. Such sessions are provided generally as group meetings with individual sessions provided on demand.

#### Prescribed exercise training

At the beginning of each training session, the patients’ symptoms were evaluated, and their blood pressure and blood sugar level were checked before being cleared to join the session. Any abnormality before or during the session would lead to its postponement.

Exercise sessions were performed using a treadmill. Each patient was directly observed by a trained nurse with a physician overseeing up to three patients at a time.

Each session lasted up to 45 min including a 5- to 10-min warm-up period (with an average speed of 3 km/h), at least 20 min of aerobic training (with speed adjusted so that the patient was at or below their target heart rate), and a 5- to 10-min cool-down period (with an average speed of 3 km/h). Sessions started short (15 to 20 min) and were progressively prolonged reaching 45 min in all patients towards the end of the program.

The target heart rate was calculated using the Karvonen formula [22] with an exercise intensity of 50 to 75%: the target heart rate = resting heart rate + (training percentage required [50–75%] × heart rate reserve); heart rate reserve = peak heart rate (estimated from the symptom-limited exercise test) − resting heart rate.

### Follow-up assessments

After completion of the 12-week cardiac rehabilitation program, each patient was re-assessed using the same methods described before for BMI, resting blood pressure, METs achieved on symptom-limited exercise testing, fasting blood sugar, lipid profile, and trans-thoracic echocardiography.

### Statistical analysis

Data were collected, revised, coded, and entered into the IBM Statistical Package for Social Science (SPSS) version 24. Data were tested for normality. Categorical data were presented as numbers and percentages while continuous data were presented as means and standard deviations. A comparison of categorical data was performed using the chi-square test while that of continuous data was performed using a paired sample *t* test. *p* value was considered significant at less than 0.05.

## Results

### Comparing both groups at baseline

#### Baseline clinical characteristics

On comparing non-obese (*n* = 58) to obese (*n* = 62) patients at baseline, it was observed that there were more females in the obese group (*p* = 0.04). More patients in the obese group were hypertensives (*p* = 0.032). However, the number of current smokers was more in the non-obese group (*p* = 0.025). There was no difference between obese and non-obese patients regarding the mean age, the proportion of diabetics, and the proportion of patients with underlying dyslipidemia (Table 1).

**Table 1:** Comparing baseline clinical, laboratory, and echocardiographic variables

Variable	Non-obese patients (*n* = 58)	Obese patients (*n* = 62)	*p* value
**Clinical characteristics at baseline**			
Age, years	54.24 ± 9.85	52.03 ± 6.48	0.313
Female gender, *n* (%)	6 (10.34%)	20 (32.25%)	0.04
Current smoker, *n* (%)	46 (79.31%)	32 (51.6%)	0.025
Hypertension, *n* (%)	16 (27.58%)	34 (54.84%)	0.032
Diabetes, *n* (%)	14 (24.13%)	24 (38.7%)	0.225
Dyslipidemia, *n* (%)	14 (24.13%)	26 (41.93%)	0.144
BMI, kg/m^2^	27.36 ± 1.65	33.75 ± 3.81	< 0.001
Systolic BP, mmHg	121.03 ± 15.08	132.42 ± 16.68	0.008
Diastolic BP, mmHg	76.38 ± 10.26	81.61 ± 9.34	0.043
METs achieved	9.74 ± 2.47	7.97 ± 2.4	0.007
**Laboratory tests at baseline**			
Total cholesterol, mg/dl	170.86 ± 41.16	181.00 ± 34.48	0.304
LDL-C, mg/dl	95.73 ± 31.51	121.63 ± 36.52	0.005
HDL-C, mg/dl	39.34 ± 8.28	38.13 ± 8.14	0.569
Triglycerides, mg/dl	148.86 ± 76.38	167.77 ± 60.63	0.291
Fasting blood sugar, mg/dl	101.48 ± 24.80	109.13 ± 31.10	0.299
**Echocardiographic parameters at baseline**			
LVEDD, mm	52.38 ± 4.74	52.16 ± 4.51	0.856
LVESD, mm	37.28 ± 5.52	35.97 ± 4.71	0.327
LVEF, %	54.45 ± 8.34	56.61 ± 9.47	0.353

Continuous variables are expressed as mean and standard deviation whereas categorical variables are expressed as number (percentage)

*BMI* body mass index, *BP* blood pressure, *METs* metabolic equivalent of tasks, *LDL-C* low-density lipoprotein cholesterol, *HDL-C* high-density lipoprotein cholesterol, *LVEDD* left ventricular end-diastolic diameter, *LVESD* left ventricular end-systolic diameter, *LVEF* left ventricular ejection fraction

Additionally, obese patients had higher baseline systolic and diastolic blood pressure (BP) recordings (*p* = 0.008, 0.043 respectively) and were able to achieve fewer METs during the baseline symptom-limited exercise test (*p* = 0.007) (Table 1).

#### Laboratory tests at baseline

LDL-C was significantly higher in obese patients (*p* = 0.005). However, there was no difference between obese and non-obese patients regarding total cholesterol, HDL-C, triglyceride levels, and fasting blood sugar (Table 1).

#### Trans-thoracic echocardiography at baseline

There was no difference between obese and non-obese patients at baseline regarding left ventricular end-diastolic dimensions, left ventricular end-systolic dimensions, and left ventricular ejection fraction (Table 1).

### Comparing both groups at the end of the cardiac rehabilitation program

On comparing non-obese to obese patients after completion of the 12-week cardiac rehabilitation program, BMI was still higher in the obese group (*p* < 0.001). Obese patients achieved less METs on a symptom-limited exercise test (*p* = 0.002). However, there was no difference between non-obese and obese patients regarding each of the systolic and diastolic BP recordings (Table 2).

**Table 2:** Comparing clinical, laboratory, and echocardiographic variables at the end of the program

Variable	Non-obese patients (*n* = 58)	Obese patients (*n* = 62)	*p* value
**Clinical characteristics at the end of the program**			
BMI, kg/m^2^	26.77 ± 1.77	31.97 ± 3.47	< 0.001
Systolic BP, mmHg	117.41 ± 7.27	120.81 ± 11.19	0.172
Diastolic BP, mmHg	76.55 ± 5.99	76.77 ± 7.91	0.903
METs achieved	11.89 ± 2.43	9.82 ± 2.59	0.002
**Laboratory tests at the end of the program**			
Total cholesterol, mg/dl	138.41 ± 29.53	151.13 ± 25.95	0.081
LDL-C, mg/dl	78.37 ± 27.34	95.86 ± 26.45	0.015
HDL-C, mg/dl	44.00 ± 8.46	43.74 ± 7.95	0.903
Triglycerides, mg/dl	122.49 ± 63.73	129.48 ± 48.44	0.633
Fasting blood sugar, mg/dl	95.62 ± 22.24	101.87 ± 23.01	0.290
**Echocardiographic parameters at the end of the program**			
LVEDD, mm	52.17 ± 4.51	52.35 ± 4.66	0.878
LVESD, mm	36.10 ± 5.09	35.84 ± 4.75	0.836
LVEF, %	56.83 ± 7.83	57.35 ± 8.95	0.809

Continuous variables are expressed as mean and standard deviation whereas categorical variables are expressed as number (percentage)

*BMI* body mass index, *BP* blood pressure, *METs* metabolic equivalent of tasks, *LDL-C* low-density lipoprotein cholesterol, *HDL-C* high-density lipoprotein cholesterol, *LVEDD* left ventricular end-diastolic diameter, *LVESD* left ventricular end-systolic diameter, *LVEF* left ventricular ejection fraction

#### Laboratory tests at the end of the program

LDL-C remained significantly higher in obese patients (*p* = 0.015). There was no difference between obese and non-obese patients regarding total cholesterol, HDL-C, triglyceride levels, and fasting blood sugar (Table 2).

#### Trans-thoracic echocardiography at the end of the program

There was no difference between obese and non-obese patients at the end of the program regarding left ventricular end-diastolic dimensions, left ventricular end-systolic dimensions, and left ventricular ejection fraction (Table 2).

### Paired delta change and comparing the magnitude of change between non-obese and obese patients at the end of the program

#### BMI

Both non-obese and obese patients showed a significant reduction in BMI compared to their baseline (*p* < 0.001 for both). However, on comparing the magnitude of change between both patient groups, BMI was more reduced in obese patients (*p* < 0.001).

#### Blood pressure

Non-obese patients had no significant change in both systolic and diastolic BP compared to their baseline, while obese patients had a significant reduction in both systolic (*p* < 0.001) and diastolic (*p* = 0.002) BP compared to their baseline.

On comparing the magnitude of change between both patient groups, both systolic (*p* = 0.016) and diastolic (*p* = 0.038) BP were more reduced in obese patients (Table 3).

**Table 3:** Paired delta change and comparing the magnitude of change between non-obese and obese patients at the end of the program

Variable	Paired delta change for non-obese patients (*n* = 58)	Paired delta change for non-obese patients, *p* value	Paired delta change for obese patients (*n* = 62)	Paired delta change for obese patients, *p* value	Comparing the magnitude of change between non-obese and obese patients, *p* value
**Clinical characteristics**					
BMI, kg/m^2^	− 0.60 ± 0.70	< 0.001	− 1.78 ± 1.46	< 0.001	< 0.001
Systolic BP, mmHg	− 3.62 ± 14.14	0.197	− 11.61 ± 10.68	< 0.001	0.016
Diastolic BP, mmHg	0.17 ± 10.13	0.928	− 4.84 ± 8.11	0.002	0.038
METs achieved	2.15 ± 1.07	< 0.001	1.85 ± 0.72	< 0.001	0.211
**Laboratory tests**					
Total cholesterol, mg/dl	− 32.46 ± 23.93	< 0.001	− 29.87 ± 18.56	< 0.001	0.641
LDL-C, mg/dl	− 17.37 ± 13.28	< 0.001	− 25.76 ± 14.19	< 0.001	0.022
HDL-C, mg/dl	4.66 ± 2.59	< 0.001	5.61 ± 1.31	< 0.001	0.073
Triglycerides, mg/dl	− 26.37 ± 34.88	< 0.001	− 38.29 ± 34.55	< 0.001	0.189
Fasting blood sugar, mg/dl	− 5.86 ± 8.05	< 0.001	− 7.26 ± 12.30	< 0.001	0.608
**Echocardiographic parameters**					
LVEDD, mm	− 0.21 ± 2.81	0.695	0.19 ± 1.87	0.569	0.516
LVESD, mm	− 1.17 ± 2.16	0.007	− 0.13 ± 1.95	0.714	0.05
LVEF, %	2.38 ± 2.93	< 0.001	0.74 ± 2.52	0.111	0.024

Continuous variables are expressed as mean and standard deviation

*BMI* body mass index, *BP* blood pressure, *METs* metabolic equivalent of tasks, *LDL-C* low-density lipoprotein cholesterol, *HDL-C* high-density lipoprotein cholesterol, *LVEDD* left ventricular end-diastolic diameter, *LVESD* left ventricular end-systolic diameter, *LVEF* left ventricular ejection fraction

#### METs achieved

Both non-obese and obese patients showed a significant increase in METs achieved on symptom-limited exercise testing compared to their baseline (*p* < 0.001 for both). However, there was no difference in comparing the magnitude of change in METs achieved between both patient groups (*p* = 0.211).

#### Laboratory tests

Both non-obese and obese patients showed a significant reduction in total cholesterol, LDL-C, triglyceride, and fasting blood sugar levels, in addition to a significant increase in HDL-C level compared to their baseline (*p* < 0.001 for all).

On comparing the magnitude of change between both patient groups, LDL-C was more reduced in obese patients (*p* = 0.022). There was no difference in comparing the magnitude of change for other laboratory tests (Table 3).

#### Trans-thoracic echocardiography

Compared to their baseline, non-obese patients had a significant reduction in left ventricular end-systolic dimensions (p = 0.007), a significant increase in left ventricular ejection fraction (*p* = 0.007) and a significant increase in left ventricular ejection fraction (*p* < 0.001), and no change in left ventricular end-diastolic dimensions. While obese patients showed no change in left ventricular end-diastolic dimensions, left ventricular end-systolic dimensions, and left ventricular ejection fraction compared to their baseline.

On comparing the magnitude of change between both patient groups, left ventricular end-systolic dimensions were more reduced while left ventricular ejection fraction was more increased in non-obese patients (Table 3).

## Discussion

Obese individuals are considered a high-risk group for the development of cardiovascular disease. Obesity itself is a risk factor for coronary artery disease and is associated with an increased incidence of metabolic syndrome, dyslipidemias, and leading a sedentary lifestyle. Cardiac rehabilitation programs lead to a significant reduction in cardiovascular death associated with improvement in body weight and functional capacity [1, 2, 9, 13]. This study aimed to compare the effect of a phase 2 cardiac rehabilitation program on obese and non-obese patients with stable coronary artery disease.

The main findings of this study are as follows: (1) obese patients had more reduction of BMI, systolic and diastolic BP, and LDL-C levels after completion of a cardiac rehabilitation program compared to non-obese patients; (2) non-obese patients had more increase in left ventricular ejection fraction at the end of the program; and (3) METs achieved were significantly improved in both groups of patients compared to their respective baseline values; however, there was no difference on comparing the magnitude of this increase between both groups at the end of the program.

Secondary findings for our study population of Egyptian patients with stable coronary artery disease are as follows: (1) obesity was more common among females, (2) smoking was more common among non-obese patients, and (3) obese patients had a higher prevalence of hypertension, higher baseline LDL-C levels, and could achieve fewer METs on a symptom-limited exercise test compared to non-obese patients.

Although participation in cardiac rehabilitation programs is recommended by international cardiovascular societies following cardiac events to improve both morbidity and mortality [10, 11] with the long-term purpose and hope of putting such patients on the right track to prevent future cardiovascular events, it has been found that long-term participation in physical activity after completion of a cardiac rehabilitation program is as low as 15 to 40% of those who completed such programs 6 months after their completion [23]. This has been reported to be even lower in obese individuals whose obesity has a negative impact on the maintenance of an exercise-based lifestyle following successful completion of a cardiac rehabilitation program [24] and that is what makes them a risk group worthy of investigation.

Since rates of weight loss after completion of cardiac rehabilitation programs remain discouraging, some researchers have called for designing cardiac rehabilitation programs specifically tailored to obese individuals with weight loss programs incorporated into them [25]. Weight loss in cardiac rehabilitation programs reported in scientific literature and the current study is generally modest with an average loss of 0.5 kg over the 3-month period of the program. Cardiac rehabilitation programs are therefore considered to make patients fitter (with significant improvement in METs achieved) but not slimmer [26].

In a study comparing 116 obese patients to 198 non-obese patients with recent cardiac events (including recent MI, acute coronary syndrome, percutaneous coronary intervention, or coronary artery bypass grafting), at baseline, the authors found that obese patients were younger, with a higher incidence of hypertension and higher total cholesterol and LDL-C levels. At the end of the program, it was reported that obese patients had more reduction of BMI, while non-obese patients had more improvement in exercise capacity (in the form of METs achieved). There were no differences in comparing changes in total cholesterol, LDL-C, and HDL-C levels between both groups of patients [27].

Another study enrolled 84 obese and 121 non-obese women with stable coronary artery disease in a cardiac rehabilitation program. At baseline, exercise capacity (in the form of METs achieved) was higher in the non-obese group. There were no differences between both groups regarding serum triglycerides, total cholesterol, LDL-C, HDL-C, and fasting blood sugar levels. At the end of the program, the only positive findings were more significant reduction in BMI and more increase in exercise capacity in the obese group. Other effects of cardiac rehabilitation were similar between both groups [28]. The same group of researchers re-examined the effects of an 8-week cardiac rehabilitation program for obese and non-obese women with stable coronary artery disease on functional capacity in the form of METs achieved and reported that although METs achieved improved in both groups compared to their baseline, there was no difference in the magnitude of increase in METs on comparing both groups at the end of the program [29].

A recent study examined the impact of body weight on expected benefits from a cardiac rehabilitation program in 731 patients—of whom 167 were obese—following an acute coronary syndrome. Obese patients were more commonly males and had a lower functional capacity; a higher prevalence of hypertension, diabetes mellitus, and dyslipidemia; higher triglyceride levels; and lower functional capacity in the form of METs achieved at baseline. At the end of the program, both groups benefited similarly with relatively more improvement in exercise capacity in obese patients [30].

The benefits of cardiac rehabilitation have been extensively covered in the scientific literature. In 130 patients who had recovered from an acute ST-segment elevation MI where patients were divided into two equal groups, a study group participating in a cardiac rehabilitation program and a control group, it was shown that LVEF significantly improved in the study group with significant improvement in exercise capacity (assessed by the New York Heart Association functional class, 6-min walking distance, and aerobic exercise time) [31].

In a study examining 449 consecutive cardiac rehabilitation patients, where 240 were obese, it was found that obese individuals were generally younger, with a higher prevalence of diabetes mellitus, hypertension, and dyslipidemia and lower exercise capacity. All patients benefited equally from the program. The authors recommend that cardiac rehabilitation programs should be tailored for obese individuals to account for the modest weight loss at the end of the program [32].

### Study limitations

This study does not come without limitations. It is a single-center study without a healthy control group. The measurement of gas exchange during exercise was not performed. The results of this study should be considered with the usual factors and barriers affecting all cardiac rehabilitation programs in mind, such as the inability to objectively monitor patient compliance to educational and lifestyle advice and the possible confounding effects of concurrent use of anti-ischemic medication.

## Conclusion

Cardiac rehabilitation programs lead to an improvement in cardiovascular disease risk factors with more reduction in BMI, BP, and LDL-C levels in obese patients compared to non-obese ones. LVEF was more increased in non-obese individuals. Exercise capacity in the form of METs achieved was equally improved in both groups.

## Data Availability

The data sets used and analyzed during the current study are available from the corresponding author on reasonable request.

## Abbreviations

BMI: Body mass index
BP: Blood pressure
HDL-C: High-density lipoprotein cholesterol
LDL-C: Low-density lipoprotein cholesterol
LVEDD: Left ventricular end-diastolic diameter
LVEF: Left ventricular ejection fraction
LVESD: Left ventricular end-systolic diameter
MET: Metabolic equivalent of tasks
MI: Myocardial infarction
SPSS: Statistical Package for Social Science
